# Distinct chromatin features characterize different classes of repeat sequences in *Drosophila melanogaster*

**DOI:** 10.1186/1471-2164-15-105

**Published:** 2014-02-06

**Authors:** Kristina Krassovsky, Steven Henikoff

**Affiliations:** 1 Basic Sciences Division, Fred Hutchinson Cancer Research Center, 1100 Fairview Avenue North, Seattle, Washington 98109, USA; 2 Molecular and Cellular Biology Program, University of Washington, Seattle, Washington 98195, USA; 3 Howard Hughes Medical institute, Fred Hutchinson Cancer Research Center, Seattle, Washington 98109, USA

**Keywords:** DNA satellites, Next-generation sequencing, ChIP-seq, Histone modification

## Abstract

**Background:**

Repeat sequences are abundant in eukaryotic genomes but many are excluded from genome assemblies. In *Drosophila melanogaster* classical studies of repeat content suggested variability between individuals, but they lacked the precision of modern high throughput sequencing technologies. Genome-wide profiling of chromatin features such as histone tail modifications and DNA-binding proteins relies on alignment to the reference genome and hence excludes highly repetitive sequences.

**Results:**

By analyzing repeat libraries, sequence complexity and k-mer counts we determined the abundances of different *D. melanogaster* repeat classes in flies in two public datasets, DGRP and modENCODE. We found that larval DNA was depleted of all repeat classes relative to adult and embryonic DNA, as expected from the known depletion of repeat-rich pericentromeric regions during polytenization of larval tissues. By applying a method that is independent of alignment to the genome assembly, we found that satellite repeats associate with distinct H3 tail modifications, such as H3K9me2 and H3K9me3 for short repeats and H3K9me1 for 359 bp repeats. Short AT-rich repeats however are depleted of nucleosomes and hence all histone modifications and associated chromatin proteins.

**Conclusions:**

The total repeat content and association of repeat sequences with chromatin modifications can be determined despite repeats being excluded from genome assemblies, revealing unexpected distinctions in chromatin features based on sequence composition.

## Background

A large fraction of almost all eukaryotic genomes consists of tandemly repeated sequences, often called satellite DNA [1]. Because satellite DNA repeat units are short and vary little if at all in sequence, they are mostly excluded from genome assemblies [2]. This is unfortunate, because some satellite sequences are known to have important functions. For example centromeres – chromosome loci that form microtubule attachment sites during mitosis - are known to be positioned on repeat sequences in many organisms [1]. Another example is telomeres – sequences that cap chromosome ends. Also, changes in satellite sequences can play roles in evolution and disease [3]. Satellite sequences might have other functions: For example, they have been shown to be important for meiotic recombination [4].

Whole genome sequencing has become a widely used tool for the discovery of genetic variation, nucleosome position, chromatin modifications and DNA binding proteins. Analysis of such experiments relies on the alignment of individual sequence segments to the reference genome. Because it is impossible to uniquely align satellite sequences they are usually excluded from the genome assembly. Thus alternative methods for analysis of repeats in sequencing data are required.

Several groups have used methods independent of the alignment to the reference genome to analyze repeat content. Parker et al. [3] used direct counting of telomere repeat sequences to estimate changes of telomere repeats in tumor cells. Hayden and Willard [5] used k-mer analysis and repeat library alignment to describe canine centromere sequences. In this study we adapt these approaches with some modifications to study the repeat content of *Drosophila melanogaster*. Drosophila is a particularly attractive model because of previous extensive characterization of its satellite repeat content by methods other than sequencing. This provides a unique opportunity to verify recovery of satellites in sequencing data.

Drosophila repeat families were initially discovered by detection of satellite bands that form during CsCl equilibrium gradient centrifugation [6,7]. When centrifuged at high force CsCl creates a gradient of Cs^+^ ions. While moving through this gradient long DNA fragments separate into distinct bands based on the buoyant density, which depends primarily on GC content. Tandem arrays of short repeat units typically have biased base composition [*e.g.* (AAGAG)_n_ is 60% A + T and 40% G + C)], so that they effectively separate from long DNA fragments of average base composition comprising single-copy DNA. The bands can then be extracted, cloned and sequenced. Three of four of such bands were shown to consist of short (5 to 10 bp) repeats, while the fourth one consisted of longer (359 bp) repeat sequences [8]. Both classes of these tandem repeats are highly abundant in the genome and map primarily to centromeric and pericentric regions of chromosomes. Another class of repeats is derived from transposable elements, found in all eukaryotic genomes. These are DNA sequences that have inserted copies of themselves into new positions in the genome, and are interspersed with single-copy or satellite sequences. Transposons have been shown to comprise ~15% of the *Drosophila melanogaster* genome [9].

Most of the repeated sequences are packaged into heterochromatin – condensed and mostly transcriptionally silent chromatin identified cytologically as being more refractile and more densely staining [10]. Heterochromatin can be divided into constitutive, chromatin that is permanently condensed and is found in pericentric and telomeric regions, and facultative, gene-containing chromatin where condensation is associated with repression of gene expression [11]. It is thought that this condensation and gene repression is achieved partly by posttranslational histone modifications, which are known to be enriched at different functional elements. For example, H3K4me3 is found at promoters of active genes [12] in a variety of organisms. In flies it has been shown that constitutive heterochromatin is associated with H3K9me2 while repressed genes in facultative heterochromatin are enriched in H3K27me3 [13].

Associations of specific DNA binding proteins with histone modifications are currently studied by chromatin immunoprecipitation followed by sequencing (Chip-Seq). Analysis of such experiments has thus far been limited to single-copy sequences and interspersed repeats. Studies of tandemly repeated sequences in heterochromatin by Chip-Seq are impeded by the inability to uniquely align repeat-containing reads to the reference genome.

Recently two large-scale initiatives generated comprehensive *D. melanogaster* sequencing datasets. One is the Drosophila Genetic Reference Panel (DGRP) which included sequencing of 200 inbred fly lines generated from wild caught flies [14]. Data generated by DGRP were used to study phenotype-genotype associations and evolution of the subset of repeat sequences that could be mapped uniquely. The other large-scale initiative is modENCODE, which included Chip-Seq experiments for a number of DNA binding proteins and histone tail modifications from different developmental stages of Drosophila. In this study we used these publicly available resources to analyze the repeat content of the *D. melanogaster* genome and to identify histone tail modifications and DNA binding proteins associated with satellites.

## Results and discussion

### Strategy for quantifying repeats

**Figure 1:**
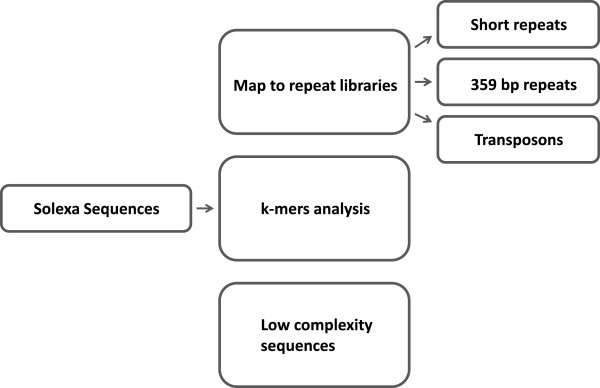
**Strategy for quantifying repeats in sequencing datasets.** Three independent approaches were used to quantify repeats: 1) map to repeat libraries; 2) count k-mers; 3) extract and analyze low complexity sequences.

We used three independent metrics to describe repeat content: (1) alignment to the libraries of known repeats; (2) estimation of the proportion of low complexity sequences; (3) classification of the most frequent k-mers (Figure 1).

Repeat libraries were constructed for short repeats (FlyBase), 359 bp repeats [15] and transposons (FlyBase) by extraction from existing genome assemblies including unassembled contigs. A complexity score similar to the DUST score used by the BLAST program to exclude low-complexity sequences was calculated for each sequenced fragment. Short repeat units have low complexity scores. This means that finding the number of sequences with a low complexity score allows us to estimate the percentage of short repeats independent of alignment programs.

Another alternative to alignment to reference libraries is k-mer analysis. A k-mer is a sequence of length k found in the sequencing dataset. For example, the 5-mer AAGAG is one of the 5-mers found in the sequence AAGAGAAGAG. By counting all k-mers we can find sequences that occur very frequently in the genome. Satellites result in k-mers that have a much higher count than the rest of the genome. K-mer and low complexity analyses provide an estimation of the completeness of repeat libraries and find abundant sequences not included in the libraries.

To find the overall fold enrichment of satellites in the ChIP-seq experiments we calculated fold enrichment of each k-mer present at least twice in both ChIP and input samples. We then grouped k-mers by their enrichment value and classified each k-mer as being in one of the repeat families, in the euchromatin, or not previously mapped, by aligning to each of the repeat libraries and the whole annotated genome. Classification and grouping of k-mers in this manner allows visualization of enrichment or depletion of a particular repeat family.

**Figure 2:**
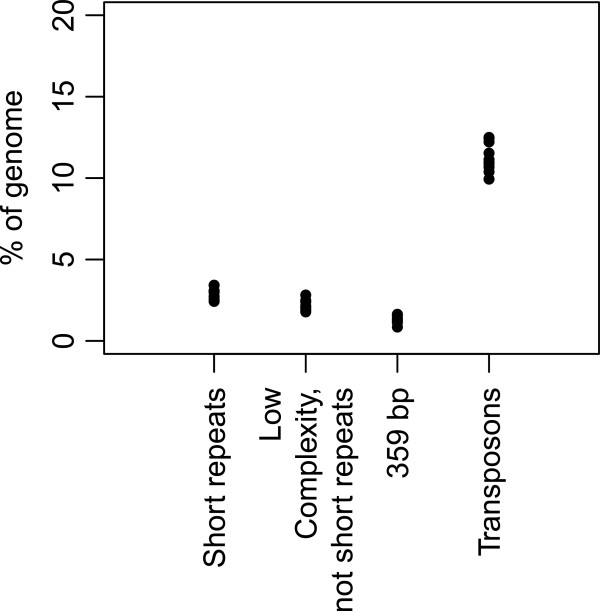
**Percentage of four repeat classes in the genomes of 10 DGRP wild-caught fly lines.** Paired-end reads from individual sequencing experiments were mapped to short repeat, 359 bp and transposon libraries and the percentage of total reads was calculated. The percentage of "low complexity, not short repeats" sequences was found by subtracting the percentage of short repeat sequences from the percentage of sequences with a low complexity score. Each value represents a median for a single fly line.

DGRP datasets include multiple sequencing runs for the same fly line, which allowed us to distinguish variability introduced by experimental variation from biologically relevant variation that occurred due to differences between individual flies in the wild population. We calculated the percentage of reads that mapped to each of the repeat families as well as the percentage of low complexity sequences (Figure 2) and averaged the percentages between experiments of the same fly line. To determine whether variation is statistically significant we performed ANOVA tests (Table 1) and found that short repeats and 359 bp repeats did not differ significantly between DGRP fly lines. Low complexity sequences and transposons changed slightly but significantly overall. These findings imply that sequence variation is confined to interspersed, but not tandem repeat sequence families.

**Table 1:** Abundance of different repeat families in DGRP flies

**Repeat family**	**% of total genome (mean between lines)**	**Standard deviation between lines**	**p-value (significance of the difference between lines)**
**Total low complexity**	5.2	0.44	6.968e-06***
**Low complexity, not short repeats**	2.21	0.32	0.19
**Short repeats**	2.98	0.33	0.03128*
**359 bp repeats**	1.31	0.24	0.01315*
**Transposons**	11.3	0.88	1.613e-05***

*p<0.1, **p<0.005, ***p<0.001.

### All repeat classes are depleted in larval relative to adult and embryonic development stages

An unusual feature of the Drosophila genome is changes in the repeat content for some cells of the organism. The best-known example is the polytene chromosomes of larval salivary glands [16,17]. During larval stages of development rapid growth of the organism requires high levels of gene expression. To efficiently accommodate this need cells undergo multiple rounds of replication without mitoses or cell divisions. This process results in banded polytene chromosomes, which are composed of multiple precisely aligned copies of sister chromatids and homologs. Chromosomes in most larval tissues are polytene, with salivary gland chromosomes being the most extremely polytenized. Polytene chromosomes are depleted of heterochromatin, especially satellite sequences. Another cell type that is depleted of satellite DNA is nurse cells, which produce yolk that is stored in the egg and consumed during embryonic development [18]. Unlike salivary glands and other larval tissues, nurse cell nuclei lack polytene structure.

**Figure 3:**
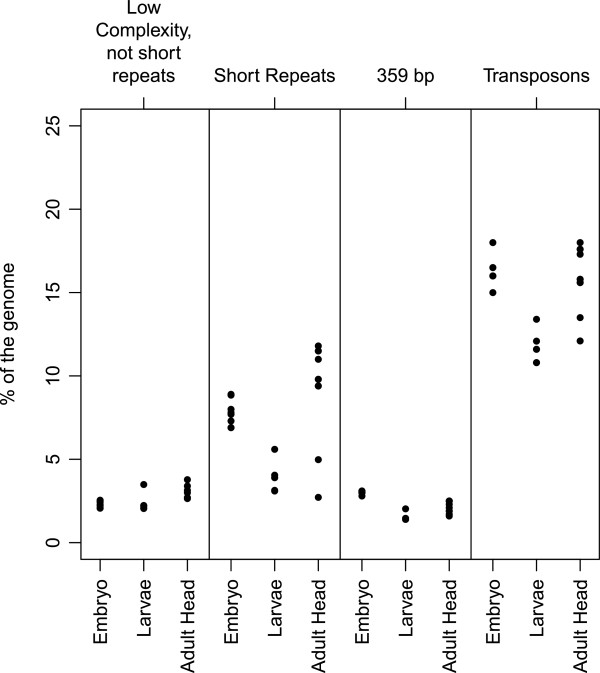
**Percentage of four repeat classes in the genomes of embryo, larvae and adult modENCODE Oregon R flies.** See the legend to Figure 2. Values for each sequencing experiment are shown, grouped by developmental stage.

We took advantage of the sequencing datasets from different developmental stages available from modENCODE. In order to determine the abundance of different repeat families in the genomes of embryos, larvae and adults, we mapped sequences from input datasets to the repeat libraries and calculated the percentage of mapped reads. We also calculated the percentages of low complexity sequences in each dataset (Figure 3). To determine whether repeat sequence abundance changes between developmental stages, we compared the median percentage abundance between sequences from the same developmental stage to that across all sequences (Table 2). As expected, we found that all repeat families are significantly depleted in larvae relative to embryos and adults (Table 3).

**Table 2:** Abundance of different repeat families in the fly genome by developmental stage (modENCODE dataset)

**Developmental stage**	**Repeat family**	**% of total genome (median)**
**Embryo (12–14 hr)**	Low complexity	10.22
	Short repeats	7.75
	359 bp repeats	3.00
	Transposons	16.0
**Larvae**	Low complexity	6.13
	Short repeats	3.90
	359 bp repeats	1.43
	Transposons	11.6
**Adult head**	Low complexity	12.45
	Short repeats	9.8
	359 bp repeats	2.20
	Transposons	15.8

**Table 3:** p-values that indicate significance of the difference between the developmental stages

**Developmental stage**	**Repeat family**	**p-value**	**Change**
**Embryo/adult**	Low complexity	0.07273	2.23
	Short repeats	0.2209	2.05
	359 bp repeats	6.087e-06***	0.8
	Transposons	0.627	0.2
**Adult/larvae**	Low complexity	0.0003176***	6.32
	Short repeats	0.001056**	5.9
	359 bp repeats	0.002137**	0.77
	Transposons	0.0002104***	4.2
**Embryo/larvae**	Low complexity	1.281e-05***	4.09
	Short repeats	1.922e-06***	3.85
	359 bp repeats	2.677e-09***	1.57
	Transposons	7.374e-07***	4.4

*p<0.1, **p<0.005, ***p<0.001.

The percentage of short repeats has been previously estimated at 5-10% [19] using cot curves and at 18-22% [7,8] using CsCl gradients for embryos of the Oregon R wild-type lab strain. In the datasets examined the short repeat content is 3% on average for DGRP flies, 12% for embryos and adult heads and 3% for larvae of modENCODE flies. The lower repeat content in DGRP samples might be explained by the use of whole flies in these experiments, where nurse and follicle cells that make up most of the mass of healthy adult female flies in uncrowded cultures [20] will lower the satellite repeat content.

Multiple experimental replicates available in both modENCODE and DGRP datasets present an opportunity to examine the reliability of modern sequencing methods for recovery of repeated sequences. On the one hand, the abundance of short repeats varies only slightly between distinct DGRP fly lines and replicate datasets derived from the same fly line. On the other hand, there is considerable variation between modENCODE replicate datasets. This variability is unlikely to be due to alignment bias because we obtained similar estimates using an alternative method of finding short repeat sequences based on sequence complexity. High variation might be due to random loss or amplification of repeats by PCR during Illumina library preparation and flow cell cluster generation. PCR is known to have biases in amplification due to composition [21]. Alternatively, variation might be due to real sequence heterogeneity among individuals of the same laboratory strain, as has been previously suggested for satellite sequences [4]. This possibility is consistent with our observation that short repeat recovery is less variable for embryos than adult flies. It is possible that fewer adult flies are needed for the recovery of material necessary for constructing Illumina sequencing libraries compared to embryos, where there are fewer cells per individual, making inter-individual heterogeneity more evident in adult than embryo samples. Another possibility is that the differences arose from the DNA preparation method used. Unlike DGRP samples where DNA was extracted from flies directly, modENCODE samples were prepared for ChIP by extraction of cross-linked chromatin. Sonication of chromatin as opposed to sonication of naked DNA might produce additional variability between the experiments.

### The most frequent k-mers in the fly genome are known short repeats and transposons

**Figure 4:**
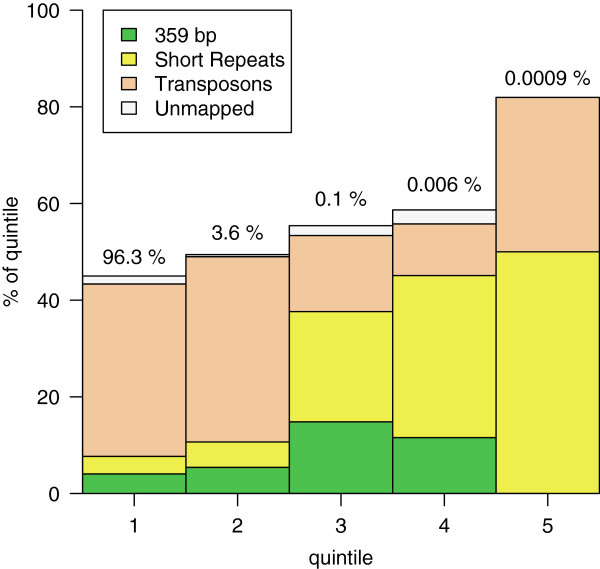
**Averaged k-mer distribution for DGRP flies grouped by repeat class.** K-mer frequencies were computed for each sequencing experiment. K-mers were split into quintiles by frequency, with quintile 5 being the most frequent. Each k-mer in the quintile was classified as mapping to short repeat, 359 bp repeat, transposon, and genome assembly or unmapped classes. The median number of k-mers that belonged to each of the repeat classes or that were unmapped for the 10 fly lines is plotted in each quintile. Numbers above indicate the percentage of the total number of k-mers falling into each quintile.

We wanted to find the most abundant repeat sequences of the fly genome independent of the mapping to the known repeat libraries. Such an estimation is important to ascertain the completeness of existing annotations and libraries. We found the occurrence of all k-mers of length 31 in all samples of DGRP. This k-mer length was chosen to be large enough to allow distinguishing unique from repeated sequences by BLAST searching (22 bp, Ref. [22]) but smaller than the minimum read length (45 bp). We then divided all k-mers of length 31 into equal quintiles based on their count and classified them by repeat family, if known. The classification of repeats averaged across all datasets of 10 DGRP fly lines is shown in Figure 4. The most frequent k-mers belong to the short repeat class. Almost all of the frequent k-mers are classified as belonging to one of the known repeat families.

**Figure 5:**
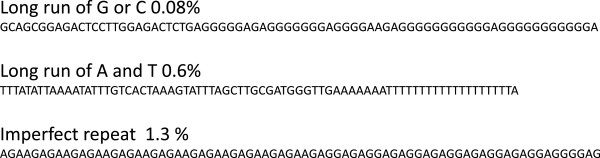
**Examples of low complexity sequences that are not classified as short repeats.** Sequences with a low complexity score were separated using Prinseq and mapped to the short repeat library. Low complexity sequences that did not map to the short repeat library were separated and some representative examples are shown.

We noticed that ~2% of the fly genome in DGRP datasets can be classified as low-complexity sequences that are not present in the short repeat library. Two-thirds of the low-complexity sequences represent imperfect short repeats, i.e. runs of short repeats interspersed with a small number of changes. By manual scrutiny we observed that many of the remaining sequences contained long runs of single nucleotides of which the large majority consisted of stretches of As or Ts, with a small percentage with stretches of Gs or Cs. Examples of such sequences together with their proportion of the total genome are presented in Figure 5. Occurrences of tracks of 'T’ and 'A’ were previously examined in several genomes [23] and found to be more abundant than expected by chance. Such sequences have been previously described near promoters of some genes and have been shown to effectively exclude nucleosomes [24].

**Figure 6:**
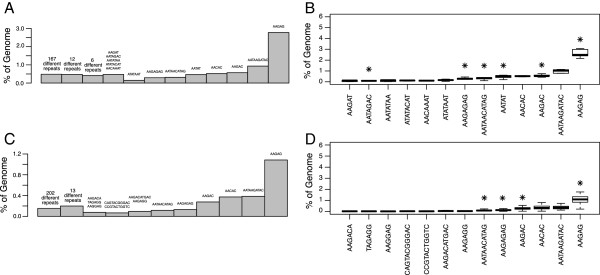
**The most frequent short repeats in the fly genome.** modENCODE **A)** and DGRP **C)** reads that mapped to a particular short repeat sequence were counted. Repeats were split into groups based on the number of reads that mapped to them. Repeat sequences in each group and the percentage of each group relative to the whole genome are indicated. **B)** and **D)** Repeats from the top groups shown in **A)** and **C)**, respectively. The percentage of total reads mapped to each repeat over multiple experiments is shown in the form of boxplots. An asterisk (*) marks each repeat group identified in previous studies based on cloning and sequencing of CsCl gradient bands.

Previous studies of fly repeats using cloning and sequencing of satellite bands isolated from CsCl gradients showed that satellite DNA in Drosophila includes 11 individual short sequences [8]. A limitation of this approach recognized by the authors is the instability of repeats when cloned into *Escherichia coli* and hence possible biases against them, as well as co-sedimentation of repeat-containing fragments with single-copy DNA. We wanted to compare this previous result with sequences obtained by modern high thoughput sequencing. To do so we counted the number of reads mapped to each sequence entry in the short repeat library in DGRP and modENCODE input datasets. We then grouped individual repeats by their frequency (Figure 6a and b). We found that although our short repeat library contains more than 200 individual repeats, only 13–14 of them make up ~90% of the short repeat satellite reads. We also identified the most abundant short repeats and compared them to the sequences identified previously by cloning and sequencing CsCl gradient bands (Figure 6c and d) [8]. Out of 11 sequences identified in previous work we found that 4 ("AATAACATAG", "AAGAGAG", "AAGAG", "AAGAC") are among the most abundant repeats in DGRP flies and 6 ("AATAACATAG", "AAGAGAG", "AAGAG", "AATAT", "AAGAC", "AATAGAC") in modENCODE flies. Interestingly, some of the short repeats differ only in the interchange of two nucleotides, such as "AATAACATAG" and "AATAAGATAC". Such sequences would have the nearly same buoyant density and will band together in a CsCl gradient.

In both DGRP and modENCODE samples we found repeat sequences that were not previously identified as abundant repeats. We might attribute this discrepancy to differences in the method of DNA extraction or to evolutionary changes that occurred in the Oregon R strain, which has been maintained in various laboratories for several decades. Abundances of specific repeat sequences among the DGRP fly lines are very similar, indicating strong homogeneity of the satellites among individual flies in the wild outbred population. We did not find four of the previously reported repeats ("AACAA", "AATAAC", "AATAC" and "AATAG") among the top repeats in modENCODE samples, although they are present at very low abundance in both modENCODE and DGRP samples.

### Histone H3 modifications are differentially associated with short repeat sequences

**Figure 7:**
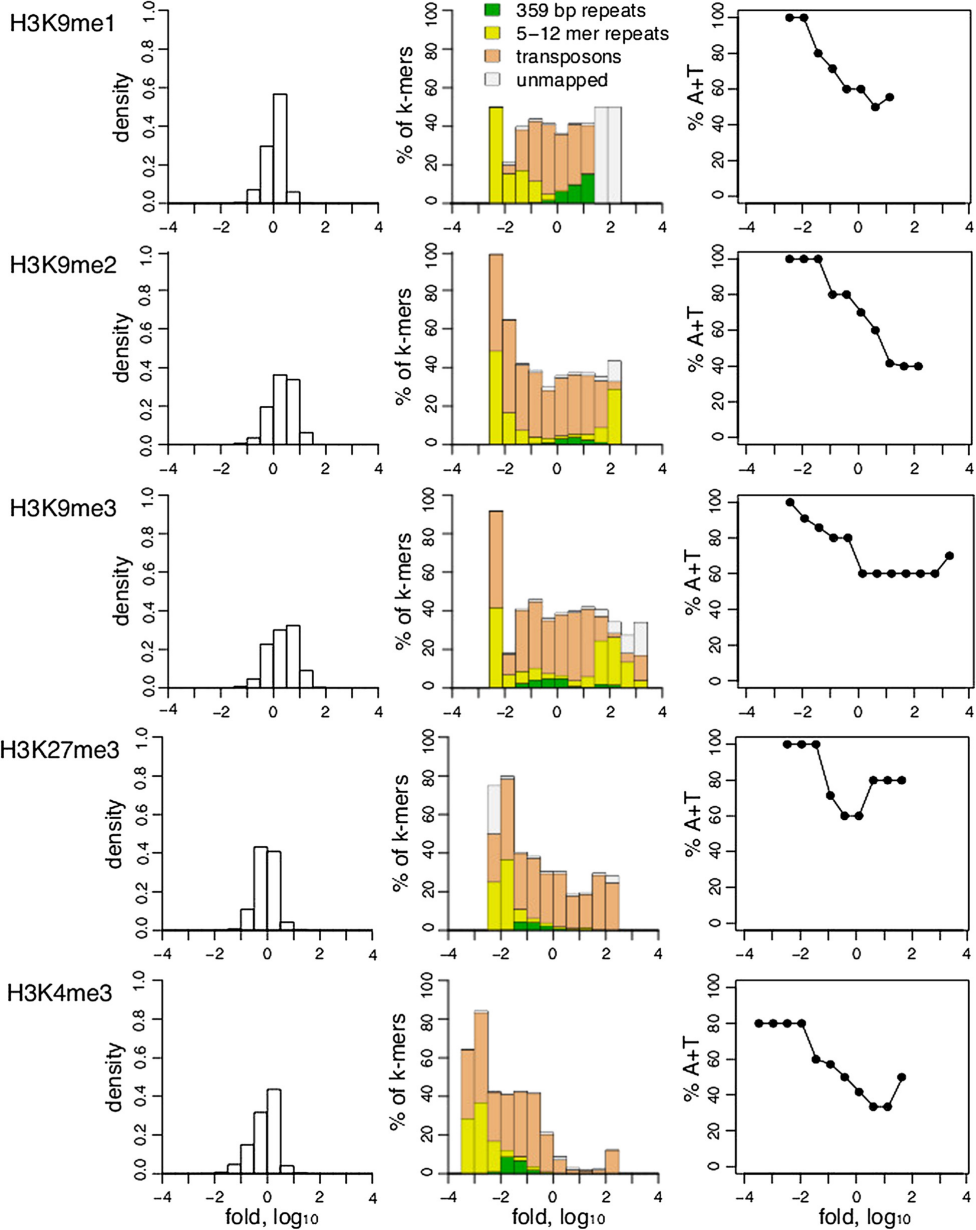
**Association of epigenetic marks with repeat classes.** For each Chip-Seq replicate k-mer frequencies were determined for both input and IP sequences. For each k-mer present in both input and IP at least twice, the enrichment of IP over input was calculated. K-mers were grouped by enrichment. Left: Distribution of counts in each group. Middle: K-mers in each group were classified as short repeats, 359 bp repeats, transposons, assembled genome or unmapped. The percentage of k-mers classified in each repeat class is shown. Some k-mers were classified as both short repeats and transposons, and they are included in both groups. Right: Percentage of A + T in short repeat k-mers. For all graphs the median between experimental replicates is shown. The number of replicates was two for all modifications.

Histone tails can have various post-translational modifications that are associated with different states of the chromatin. For example, di- and tri-methylated H3K9 are known markers of heterochromatin, trimethylated H3K27 is found at facultatively silenced genes, and trimethylated H3K4me3 is associated with gene activity. All of these marks have been studied in the mappable single-copy segments of the genome. We wanted to investigate associations of these marks with different classes of repeated sequences. To do so we identified k-mers that are present in both input and IP samples obtained from embryos in the modENCODE datasets and for each k-mer we calculated its enrichment relative to the input. We then separated these k-mers into groups based on their fold enrichment (Figure 7 left). As expected, the distribution of k-mer enrichment resembles a normal distribution, with a majority of the sequences neither enriched nor depleted. We then mapped k-mer groups to each of our repeat libraries or to the genome assembly. In this way each k-mer was classified as either one of the repeat types, part of the genome assembly, or unmapped. We then plotted the percentage of each repeat type in each group as well as the percentage of unmapped k-mers in each group (Figure 7 middle). Such k-mer classification allows a visualization of enrichment of particular histone modifications in each repeat class. As expected, H3K4me3 is virtually absent from all the repeat types and H3K9me3 and H3K9me2 are enriched for some short repeats and transposons. Surprisingly, we found H3K9me1 to be depleted from short repeats but enriched in the 359 bp repeats. H3K9me1 has been shown to be a substrate for a histone methyltransferase that catalyzes di- and tri-methylation in mouse and Arabidopsis [25,26], but the specificity of chromatin association of this modification in Drosophila has not been reported previously. H3K27me3 is depleted from short and 359 bp repeats but enriched in transposons, which is consistent with it being a mark of facultative heterochromatin. As described below, some short repeats are also depleted for all histone modifications examined.

Posttranslational histone tail modifications are known to be involved in transposon silencing. Transposons are classified into groups based on their structure and mechanism of transposition. Retrotransposons, which mobilize via an RNA intermediate, are further divided into LTR (long terminal repeats) and non-LTR classes. Previous studies investigated whether some transposon families are preferentially associated with specific histone modifications. For example, a screen of 100 transposon sequences by microarray analysis found that retrotransposons have higher enrichment in H3K9me2 than other elements [11]. In contrast, *roo* retrotransposons, which are abundant in euchromatin, were found to have lower H3K9me2 association. Four families of LTR retrotransposons (*roo*, *tirant*, *412* and *F*) were also screened for preferential association with H3K9me2 and H3K27me3 in different strains of *D. melanogaster* and were found to have large variations in enrichment between the strains. However, our systematic investigation based on classification of Illumina sequencing reads both by k-mer analysis and direct counting of reads mapped to different transposon groups detected no preferential association of LTR, non-LTR or IR transposon classes with histone modifications (Additional file 1: Table S1).

### All three HP1 proteins localize to transposons

**Figure 8:**
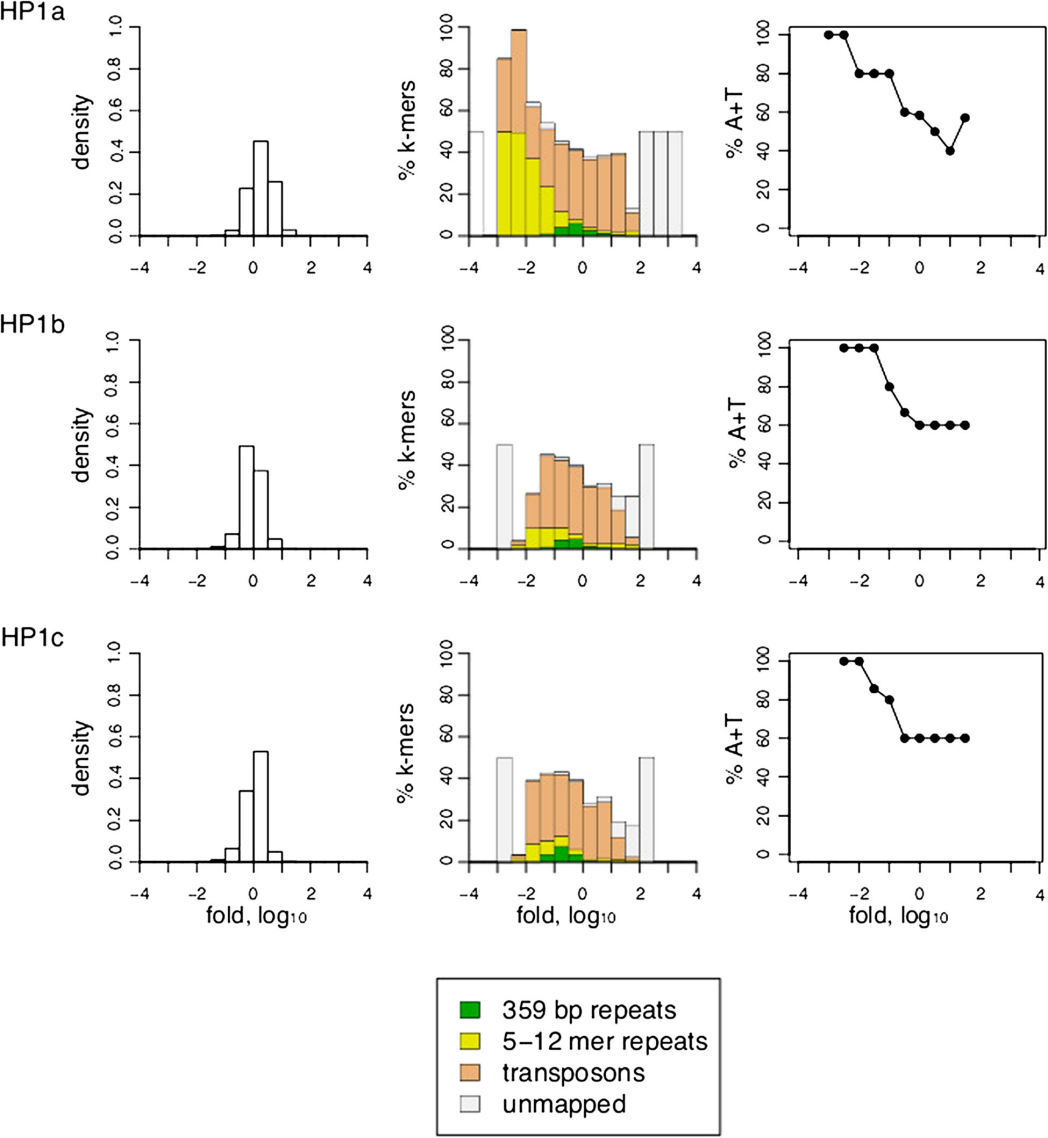
**Association of HP1 proteins with repeat classes.** Same analysis as in Figure 7 but for HP1 proteins. The number of replicates was 4 for HP1a, 1 for HP1c and 2 for HP1b.

We also examined ChIP datasets of Heterochromatin-associated Protein 1 (HP1) for association with different repeat classes. HP1 has been implicated in the formation of heterochromatin by the binding of its "chromodomain" to di- and tri-methylated H3K9 [27,28] and by dimerization of its "chromo-shadow" domain, bringing neighboring nucleosomes together to condense chromatin [29]. Drosophila has three closely related HP1 proteins, HP1a, HP1b and HP1c, each of which has been shown to have a different localization pattern by cytology [30]. HP1a has been shown to be required for silencing of transposons and is exclusively localized to heterochromatin [31,32]. HP1c localizes to euchromatin and HP1b localizes to both euchromatin and heterochromatin. However, k-mer analysis shows that all three of the HP1 proteins are enriched in transposons and depleted in other types of repeats (Figure 8 middle). This is unexpected because HP1a has not been shown to have preferential localization with different classes of heterochromatin, such as transposons versus satellites.

### AT-rich repeats are depleted of nucleosomes

We noticed that even for the H3K9me3 and H3K9me2 heterochromatic marks that are enriched in short repeats a few specific repeat sequences are depleted of these marks. This prompted us to look for a common property of short repeats that are depleted of heterochromatic marks and HP1 proteins. We first classified each repeat by the length of the repeat unit but detected no consistent trends. However, when we classified repeats by AT content, we observed that the short repeats that are depleted of HP1 family proteins and histone modifications are also very AT rich (Figures 7 and 8 right panels; Additional file 1: Table S2).

**Figure 9:**
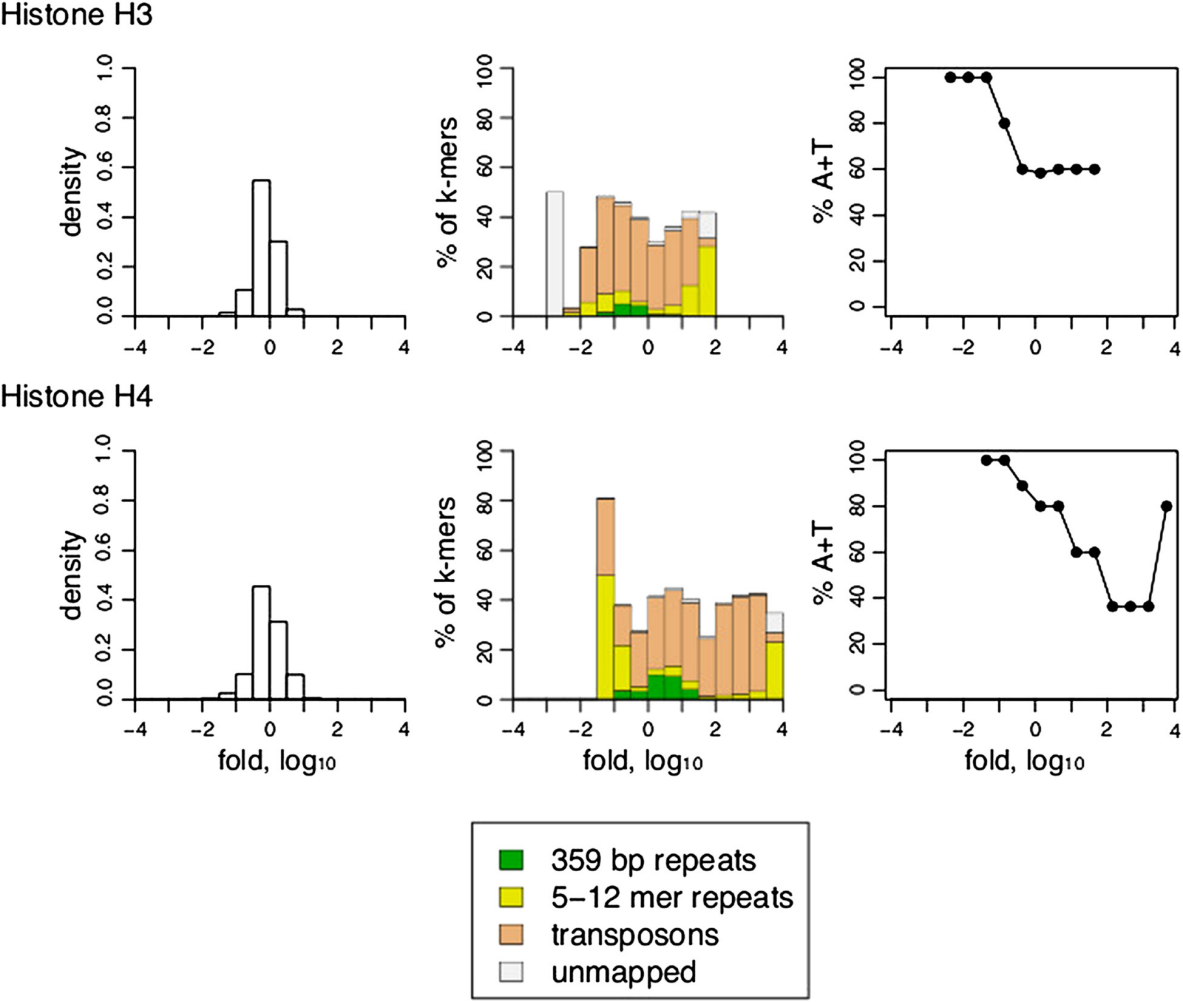
**Association of histones H3 and H4 with repeat classes.** Same analysis as in Figure 7 but for H3 and H4 histones. The number of replicates was 2 for both H3 and H4.

We hypothesized that the consistent depletion of short AT-rich repeats from ChIP datasets of histone modifications and chromatin proteins that bind them is due to the depletion of nucleosomes themselves. To test this possibility we performed k-mer analysis on sequences enriched by ChIP-seq of H3 and H4 histones. We found that these histones are also depleted of short AT-rich repeats (Figure 9; Additional file 1: Table S2). Hence depletion of histone modifications and HP1 proteins from AT-rich short repeat sequences is not due to selectivity against these chromatin features but rather is explained by the overall depletion of nucleosomes from AT-rich short repeats.

Highly AT-rich DNA has a narrow minor groove and reduced flexibility, which disfavors the tight wrapping of the double helix around the nucleosome core and results in preferential exclusion of nucleosomes [24,33]. As the (AATAT)n, (AATATAT)n and other long arrays of pure AT sequences are predicted to be especially stiff [34], they would be expected to prevent nucleosome formation. Alternatively, nucleosomes might be actively excluded by competing DNA-binding proteins. For example, D1 protein is a highly abundant nuclear protein that is preferentially bound to the narrow minor groove of AATAT *Drosophila* satellite arrays [35,36]. With ~1 D1 protein per 10 nucleosomes, and ~0.7% of the genome consisting of AATAT-containing satellites, there is enough chromatin-bound D1 to occupy ~1/2 of all the AATAT sites [(1 D1/10 nucleosomes)/(30 AATAT sites in a 150 bp span) = 0.0033% of the genome]. These alternative possibilities are not mutually exclusive, as expansion of an AATAT array would both exclude nucleosomes and promote D1 binding, consistent with the possibility that D1 protein has evolved to package stiff AT-rich satellites.

## Conclusions

We have shown that enrichment of repeated sequences can be quantified in Chip-Seq experiments despite being largely excluded from genome assemblies. The strategy of calculating k-mer enrichment relative to the input allows direct comparison of repeat sequences to single-copy regions of the genome. The strategy presented here can be applied to study other chromatin features known to be located in heterochromatin, for example centromeres.

We also have presented the first analysis of the chromatin landscape of repeat sequences in a genome-wide context. Different heterochromatic regions of *D. melanogaster* have distinct chromatin features. Satellite sequences associate with specific histone modifications such as H3K9me2 and H3K9me3. All three HP1 homologues are enriched at transposons and do not show preferences for particular types of transposons. AT-rich short repeats are depleted of nucleosomes and hence all histone modifications. We conclude that ChIP-seq datasets can be mined to provide unexpected insights into chromatin landscapes of repetitive sequences.

## Methods

### Datasets

modENCODE datasets listed in Table 4 were downloaded from http://data.modencode.org. DGRP datasets listed in Table 5 were downloaded from http://www.ncbi.nlm.nih.gov/sra?term=DGRP. For each fly line only sequences generated by the Illumina platform were used.

**Table 4:** **modENCODE datasets (**http://data.modencode.org**) used in this study**

**Assay factor**	**Development stage**	Experiment ID*
**H3K9me1**	Embryo	4123
**H3K9me2**	Embryo	4940
**H3K9me3**	Embryo	4939
**H3K4me3**	Embryo	5096
**H3K27me3**	Embryo	3955
**H3**	Embryo	5079
**H4**	Embryo	5107
**HP1a**	Embryo	3956
**HP1b**	Embryo	5111
**HP1c**	Embryo	5587
**H3K9me2**	Larvae	4958
**H3K9me3**	Larvae	4952
**H3K4me3**	Larvae	5097
**H3K27me3**	Larvae	5089
**HP1a**	Larvae	4936
**HP1b**	Larvae	5110
**HP1c**	Larvae	5112
**H3K9me2**	Adult	5259
**H3K9me3**	Adult	4933
**H3K4me3**	Adult	5098
**H3K27me3**	Adult	5583
**HP1a**	Adult	5592
**HP1b**	Adult	5590
**HP1c**	Adult	5591

*Each experiment contains both input and IP sequences and one or more replicates.

**Table 5:** DGRP datasets used in the study

**Fly line**	**Experiment ID**
**DGRP-313**	SRR018517, SRR018518, SRR018519
**DGRP-357**	SRR018285, SRR018286
**DGRP-358**	SRR018574, SRR018575, SRR029943, SRR034277, SRR034278
**DGRP-362**	SRR029164, SRR029166
**DGRP-365**	SRR018579, SRR034281, SRR034282, SRR034283
**DGRP-375**	SRR018287, SRR018288, SRR018289, SRR018290, SRR018291
**DGRP-379**	SRR018582, SRR018583, SRR018584
**DGRP-380**	SRR018591, SRR018592, SRR018593
**DGRP-391**	SRR018292, SRR018293, SRR018294, SRR060098
**DGRP-399**	SRR018295, SRR018296, SRR018297

### Repeat libraries

The short repeats library was downloaded from http://hgdownload.cse.ucsc.edu/goldenPath/dm3/bigZips/chromTrf.tar.gz. It was converted to a fasta file format and purged of duplicate entries. The 359 bp library was the one produced in [15] and obtained directly from Dr. Gustavo Kuhn.

The transposon library was downloaded from FlyBase r5.48 ftp://ftp.flybase.net/genomes/Drosophila_melanogaster/dmel_r5.48_FB2012_06/fasta/dmel-alltransposon-r5.48.fasta.gz.

### Determining repeat abundances

Sequences were mapped to a short repeat and 359 bp repeat library using BWA [37] and to transposons using Novoalign ( http://www.novocraft.com). The number of sequences mapped to the library was divided by the total number of sequences to find the percentage abundance.

### K-mer analysis

K-mers were obtained using Jellyfish [38] with the command "jellyfish count -m 31 -o output -c 3 -s 10000000 -t 12 -L 2". K-mers were split into quintiles using a custom script and aligned to repeat libraries using BWA (short repeats and 359 bp repeats) and Novoalign (transposons).

### Finding low complexity sequences

The percentage of low complexity sequences was found by running Prinseq [39] with the command "perl prinseq-lite.pl -fastq FileName.fastq -verbose -graph_data -out_good null -lc_method dust -lc_threshold 7". This command separates sequences with a complexity score above 7 and records that number in the log file.

### K-mer analysis of the ChIP-seq datasets

A k-mer count table was constructed for both Input and ChIP samples using Jellyfish and then merged using a custom R script. For each k-mer, enrichment was calculated by dividing the number of counts in the ChIP dataset by the number of counts in the corresponding Input dataset and normalized by multiplying by the ratio of the total number of sequences in input and ChIP samples. K-mers then were split into 16 groups based on enrichment. K-mer sequences from each group were aligned to repeat libraries and the genome assembly using BWA and Novoalign. The number of k-mers in each group mapped to a particular library was noted and then plotted using an R script. For experiments with two replicates the median number of k-mers in each bin is shown.

## Abbreviations

DGRP: (Drosophila Genetic Reference Panel); modENCODE: (**mod**el organism **ENC**yclopedia **O**f **D**NA **E**lements); ChIP: (chromatin immunoprecipitation); HP1: (Heterochromatin-associated protein 1).

## Competing interests

The authors declare that they have no competing interests.

## Authors’ contributions

KK and SH conceived of and designed the study. KK performed the experiments and analyses and drafted the manuscript. Both authors read and approved the final manuscript.

## Supplementary Material

Additional file 1: Table S1 Total enrichment of histone tail modifications by transposon family group. Percentage of reads mapped to each of the groups was calculated in both input and IP datasets. Enrichment was calculated as the ratio of percentage of reads in the Input dataset to Ip dataset. Values were averaged between experiment replicates and mean values together with standard deviations are presented in the table. **Table S2.** Sequences of 5-12 mer repeats identified as enriched (top 4 bins) or depleted (bottom 4 bins) in histone modifications IP samples. Only the most abundant sequences (top 90% of k-mer from each group) are shown for brevity. Percentage indicates portion of the k-mers from selected bins that map to particular sequence. Click here for file
