# Supplementary material for: Distinct chromatin features characterize different classes of repeat sequences in Drosophila melanogaster
Source: BMC Genomics. 2014 Feb 6;15:105. doi: 10.1186/1471-2164-15-105 (PMC3922421; doi:10.1186/1471-2164-15-105)
Supplement: Additional file 1: Table S1 — Total enrichment of histone tail modifications by transposon family group. Percentage of reads mapped to each of the groups was calculated in both input and IP datasets. Enrichment was calculated as the ratio of percentage of reads in the Input dataset to Ip dataset. Values were averaged between experiment replicates and mean values together with standard deviations are presented in the table. Table S2. Sequences of 5-12 mer repeats identified as enriched (top 4 bins) or depleted (bottom 4 bins) in histone modifications IP samples. Only the most abundant sequences (top 90% of k-mer from each group) are shown for brevity. Percentage indicates portion of the k-mers from selected bins that map to particular sequence. [file 1471-2164-15-105-S1.docx]

**Supplemental Table 1.** Total enrichment of histone tail modifications by transposon family group. Percentage of reads mapped to each of the groups was calculated in both input and IP datasets. Enrichment was calculated as the ratio of percentage of reads in the Input dataset to Ip dataset. Values were averaged between experiment replicates and mean values together with standard deviations are presented in the table.

| **Modification** | **LTR** | **Non LTR** | **SINE** | **IR** |
| --- | --- | --- | --- | --- |
| **H3K9me1** | 0.91 ± 0.02 | 1.04 ± 0.01 | 0.77 ± 0.08 | 1.06 ± 0.03 |
| **H3K9me2** | 2 ± 0.07 | 2.23 ± 0.1 | 2.5 ± 0.13 | 1.79 ± 0.06 |
| **H3K9me3** | 1.82 ± 0.01 | 2.18 ± 0.01 | 2.79 ± 0.1 | 1.76 ± 0.01 |
| **H3K4me3** | 0.31 ± 0.19 | 0.41 ± 0.23 | 0.36 ± 0.19 | 0.35 ± 0.24 |
| **H3K27me3** | 0.72 ± 0.04 | 0.79 ± 0.02 | 0.72 ± 0.04 | 0.75 ± 0.02 |

**Supplemental Table 2.** Sequences of 5-12 mer repeats identified as enriched (top 4 bins) or depleted (bottom 4 bins) in histone modifications IP samples. Only the most abundant sequences (top 90% of k-mer from each group) are shown for brevity. Percentage indicates portion of the k-mers from selected bins that map to particular sequence.

| **Chromatin feature** | **Enriched sequences** | **%** | **Depleted sequences** | **%** |
| --- | --- | --- | --- | --- |
| **H3K9me1** | GGTCCCGTACTC | 26.96 | AATAAGATAC | 30.34 |
|  | CAGTACGGGAC | 16.94 | AATAT * | 19.45 |
|  | CCGTACTGGTC | 16.28 | AATAACATAG * | 11.63 |
|  | AAGAG * | 5.95 | AATAC * | 5.79 |
|  | AGTACGGAACCG | 4.78 | AAGAT | 4.08 |
|  | ACAAC | 4.54 | TGTAT | 3.75 |
|  | CCTCT | 4.28 | ATATAAT | 2.96 |
|  | AAGAGAG * | 1.51 | AATAG * | 2.69 |
|  | AAGAC * | 1.21 | ATATAATA | 2.22 |
|  | CAAACACAAACA | 1.17 | AATAGAC * | 1.62 |
|  | GTACGGGACCGA | 1.03 | TAATAAA | 1.48 |
|  | CGTACTCGGTTC | 0.87 | ATATATAA | 1.44 |
|  | TGCTGCTGC | 0.78 | ATATTTT | 1.34 |
|  | GGAACCAGTAC | 0.76 |  |  |
|  | AAGAGAGAAGAG | 0.65 |  |  |
|  | GACAC | 0.64 |  |  |
|  | AAGACATGAC | 0.62 |  |  |
|  | CAAGG | 0.62 |  |  |
| **H3K9me2** | AAGAG * | 33.59 | AATAAGATAC | 48.88 |
|  | AAGAGAG * | 12.14 | AATAACATAG * | 17.92 |
|  | CCTCT | 9.32 | AATAT * | 10.64 |
|  | ACAAC | 7.35 | AATAC * | 8.16 |
|  | AAGACA | 3.85 |  |  |
|  | AAGAA | 3.76 |  |  |
|  | AAGACATGAC | 3.42 |  |  |
|  | AAGAC * | 3.42 |  |  |
|  | CTTCTC | 3.08 |  |  |
|  | TCTTCTCTTT | 2.39 |  |  |
|  | AAGAGAGAAGAG | 2.14 |  |  |
|  | TCTTTG | 1.88 |  |  |
|  | CAGTACGGGAC | 1.45 |  |  |
|  | AGAAAG | 1.37 |  |  |
| **H3K9me3** | AAGAG * | 34.53 | AATAT * | 78.43137 |
|  | AAGAGAG * | 12.48 | TAATAAA | 15.68627 |
|  | ACAAC | 7.56 | TTTATTTA | 5.882353 |
|  | CCTCT | 6.85 |  |  |
|  | AAGACA | 3.95 |  |  |
|  | AAGAA | 3.87 |  |  |
|  | AAGACATGAC | 3.51 |  |  |
|  | AAGAC * | 3.51 |  |  |
|  | CTTCTC | 3.16 |  |  |
|  | TCTTCTCTTT | 2.46 |  |  |
|  | AAGAGAGAAGAG | 2.2 |  |  |
|  | TCTTTG | 1.93 |  |  |
|  | CAGTACGGGAC | 1.49 |  |  |
|  | AGAAAG | 1.41 |  |  |
| **H3K27me3** | AAGAG * | 34.53 | AATAT * | 34.18 |
|  | AAGAGAG * | 12.48 | AATAAGATAC | 29.11 |
|  | ACAAC | 7.56 | AAGAT | 13.08 |
|  | CCTCT | 6.85 | AATAACATAG * | 8.44 |
|  | AAGACA | 3.95 |  |  |
|  | AAGAA | 3.87 |  |  |
|  | AAGACATGAC | 3.51 |  |  |
|  | AAGAC * | 3.51 |  |  |
|  | CTTCTC | 3.16 |  |  |
|  | TCTTCTCTTT | 2.46 |  |  |
|  | AAGAGAGAAGAG | 2.2 |  |  |
|  | TCTTTG | 1.93 |  |  |
|  | CAGTACGGGAC | 1.49 |  |  |
|  | AGAAAG | 1.41 |  |  |
| **H3K4me3** | AAGAG * | 34.53 | AATAAGATAC | 50.37 |
|  | AAGAGAG * | 12.48 | AATAACATAG * | 18.47 |
|  | ACAAC | 7.56 | AATAC * | 8.41 |
|  | CCTCT | 6.85 | AATAT * | 8 |
|  | AAGACA | 3.95 |  |  |
|  | AAGAA | 3.87 |  |  |
|  | AAGACATGAC | 3.51 |  |  |
|  | AAGAC * | 3.51 |  |  |
|  | CTTCTC | 3.16 |  |  |
|  | AGAAGAGAAA | 2.46 |  |  |
|  | AAGAGAGAAGAG | 2.2 |  |  |
|  | TCTTTG | 1.93 |  |  |
|  | CAGTACGGGAC | 1.49 |  |  |
|  | AGAAAG | 1.41 |  |  |
| **HP1a** | AAGAG * | 32 | AATAAGATAC | 38.39 |
|  | AAGAGAG * | 11.56 | AATAT * | 19.83 |
|  | CCTCT | 11.4 | AATAACATAG * | 14.31 |
|  | ACAAC | 7 | AATAC * | 6.19 |
|  | AAGACATGAC | 4.4 | TGTAT | 4.37 |
|  | AAGAA | 3.75 | ATATAAT | 3.03 |
|  | AAGACA | 3.66 | ATATAATA | 2.91 |
|  | AAGAC * | 3.58 |  |  |
|  | CTTCTC | 2.93 |  |  |
|  | AGAAGAGAAA | 2.28 |  |  |
|  | AAGAGAGAAGAG | 2.04 |  |  |
|  | TCTTTG | 1.79 |  |  |
|  | CTTGTCATGT | 1.55 |  |  |
| **HP1b** | AAGAG * | 32 | AATAAGATAC | 38.32 |
|  | AAGAGAG * | 11.56 | AATAT * | 19.98 |
|  | CCTCT | 11.4 | AATAACATAG * | 14.29 |
|  | ACAAC | 7 | AATAC * | 6.17 |
|  | AAGACATGAC | 4.4 | TGTAT | 4.36 |
|  | AAGAA | 3.75 | ATATAAT | 3.03 |
|  | AAGACA | 3.66 | ATATAATA | 2.91 |
|  | AAGAC * | 3.58 |  |  |
|  | CTTCTC | 2.93 |  |  |
|  | AGAAGAGAAA | 2.28 |  |  |
|  | AAGAGAGAAGAG | 2.04 |  |  |
|  | TCTTTG | 1.79 |  |  |
|  | CTTGTCATGT | 1.55 |  |  |
|  | CAGTACGGGAC | 1.38 |  |  |
| **HP1c** | AAGAG * | 32 | AATAAGATAC | 38.29 |
|  | AAGAGAG * | 11.56 | AATAT * | 20.02 |
|  | CCTCT | 11.4 | AATAACATAG * | 14.28 |
|  | ACAAC | 7 | AATAC * | 6.17 |
|  | AAGACATGAC | 4.4 | TGTAT | 4.36 |
|  | AAGAA | 3.75 | ATATAAT | 3.02 |
|  | AAGACA | 3.66 | ATATAATA | 2.9 |
|  | AAGAC * | 3.58 |  |  |
|  | CTTCTC | 2.93 |  |  |
|  | AGAAGAGAAA | 2.28 |  |  |
|  | AAGAGAGAAGAG | 2.04 |  |  |
|  | TCTTTG | 1.79 |  |  |
|  | CTTGTCATGT | 1.55 |  |  |
|  | CAGTACGGGAC | 1.38 |  |  |
| **H3** | AAGAG * | 32 | AATAAGATAC | 37.66 |
|  | AAGAGAG * | 11.56 | AATAT * | 20.35 |
|  | CCTCT | 11.4 | AATAACATAG * | 14.04 |
|  | ACAAC | 7 | AATAC * | 6.07 |
|  | AAGACATGAC | 4.4 | TGTAT | 4.28 |
|  | AAGAA | 3.75 | ATATAAT | 2.97 |
|  | AAGACA | 3.66 | ATATAATA | 2.86 |
|  | AAGAC * | 3.58 |  |  |
|  | CTTCTC | 2.93 |  |  |
|  | AGAAGAGAAA | 2.28 |  |  |
|  | AAGAGAGAAGAG | 2.04 |  |  |
|  | TCTTTG | 1.79 |  |  |
|  | CTTGTCATGT | 1.55 |  |  |
|  | CAGTACGGGAC | 1.38 |  |  |
| **H4** | GGTCCCGTACTC | 26.96 | AATAAGATAC | 30.44 |
|  | CAGTACGGGAC | 16.94 | AATAT * | 19.38 |
|  | CCGTACTGGTC | 16.28 | AATAACATAG * | 11.66 |
|  | AAGAG * | 5.95 | AATAC * | 5.81 |
|  | AGTACGGAACCG | 4.78 | AAGAT | 4.09 |
|  | ACAAC | 4.54 | TGTAT | 3.76 |
|  | CCTCT | 4.28 | ATATAAT | 2.97 |
|  | AAGAGAG * | 1.51 | AATAG * | 2.7 |
|  | AAGAC * | 1.21 | ATATAATA | 2.23 |
|  | CAAACACAAACA | 1.17 | AATAGAC * | 1.63 |
|  | GTACGGGACCGA | 1.03 | TAATAAA | 1.49 |
|  | CGTACTCGGTTC | 0.87 | ATATATAA | 1.44 |
|  | TGCTGCTGC | 0.78 | ATATTTT | 1.35 |
|  | GGAACCAGTAC | 0.76 |  |  |
|  | AAGAGAGAAGAG | 0.65 |  |  |
|  | GACAC | 0.64 |  |  |
|  | AAGACATGAC | 0.62 |  |  |
|  | CAAGG | 0.62 |  |  |
